# Preassembled Cas9 Ribonucleoprotein-Mediated Gene Deletion Identifies the Carbon Catabolite Repressor and Its Target Genes in Coprinopsis cinerea

**DOI:** 10.1128/aem.00940-22

**Published:** 2022-11-14

**Authors:** Manish Pareek, Botond Hegedüs, Zhihao Hou, Árpád Csernetics, Hongli Wu, Máté Virágh, Neha Sahu, Xiao-Bin Liu, László Nagy

**Affiliations:** a Institute of Biochemistry, Biological Research Centre, Szeged, Hungary; Royal Botanic Gardens

**Keywords:** carbon catabolite repression, genome editing, mushroom-forming fungi, CAZymes, transcription factors (TFs), split-marker, transporters

## Abstract

Cre1 is an important transcription factor that regulates carbon catabolite repression (CCR) and is widely conserved across fungi. The *cre1* gene has been extensively studied in several Ascomycota species, whereas its role in gene expression regulation in the Basidiomycota species remains poorly understood. Here, we identified and investigated the role of *cre1* in Coprinopsis cinerea, a basidiomycete model mushroom that can efficiently degrade lignocellulosic plant wastes. We used a rapid and efficient gene deletion approach based on PCR-amplified split-marker DNA cassettes together with *in vitro* assembled Cas9-guide RNA ribonucleoproteins (Cas9 RNPs) to generate *C. cinerea cre1* gene deletion strains. Gene expression profiling of two independent *C. cinerea cre1* mutants showed significant deregulation of carbohydrate metabolism, plant cell wall degrading enzymes (PCWDEs), plasma membrane transporter-related and several transcription factor-encoding genes, among others. Our results support the notion that, like reports in the ascomycetes, Cre1 of *C. cinerea* orchestrates CCR through a combined regulation of diverse genes, including PCWDEs, transcription factors that positively regulate PCWDEs, and membrane transporters which could import simple sugars that can induce the expression of PWCDEs. Somewhat paradoxically, though in accordance with other Agaricomycetes, genes related to lignin degradation were mostly downregulated in *cre1* mutants, indicating they fall under different regulation than other PCWDEs. The gene deletion approach and the data presented here will expand our knowledge of CCR in the Basidiomycota and provide functional hypotheses on genes related to plant biomass degradation.

**IMPORTANCE** Mushroom-forming fungi include some of the most efficient lignocellulosic plant biomass degraders. They degrade dead plant materials by a battery of lignin-, cellulose-, hemicellulose-, and pectin-degrading enzymes, the encoding genes of which are under tight transcriptional control. One of the highest-level regulations of these metabolic enzymes is known as carbon catabolite repression, which is orchestrated by the transcription factor Cre1, and ensures that costly lignocellulose-degrading enzyme genes are expressed only when simple carbon sources (e.g., glucose) are not available. Here, we identified the Cre1 ortholog in a litter decomposer Agaricomycete, Coprinopsis cinerea, knocked it out, and characterized transcriptional changes in the mutants. We identified several dozen lignocellulolytic enzyme genes as well as membrane transporters and other transcription factors as putative target genes of *C. cinerea cre1*. These results extend knowledge on carbon catabolite repression to litter decomposer Basidiomycota.

## INTRODUCTION

Carbon catabolite repression (CCR) is a gene regulatory mechanism that inhibits the utilization of complex carbon sources in the presence of preferred carbon sources, such as glucose (1, 2). The transcription factor Cre1 and its orthologs have been identified as key regulators of CCR and are extensively studied in several model fungal species, such as Aspergillus spp. (CreA), Saccharomyces cerevisiae (Mig1), and Neurospora crassa (Cre-1) (3–5). In the presence of glucose, Cre-1 binds promoters and represses the transcription of genes responsible for the utilization of alternate carbon sources (6–8). The absence of the preferred carbon source can lead to phosphorylation of CreA and a change in its subcellular localization, ultimately leading to derepression of its target genes involved in the utilization of nonpreferred carbon sources such as lignocellulosic plant remnants (9–12). It has been shown that manipulating the carbon catabolite repressor has an impact beyond CCR on amino acid metabolism, intracellular trehalose levels, or stress, among others (12), suggesting that Cre1/CreA/Cre-1 can directly or indirectly regulate a large suite of metabolic and other genes.

Carbon catabolite repression is best studied in the Ascomycota (2, 12, 13), whereas in the Basidiomycota several details of the process are poorly known, including upstream regulators and downstream target genes of Cre1. CCR has been demonstrated in several wood-decay fungi, including the white rotters Pleurotus ostreatus (14, 15), Dichomitus squalens (16), Ganoderma lucidum (11), Phanerochaete chrysosporium (17), and the brown rot fungus Rhodonia placenta (18). This suggests that orthologs of Cre-1 should exist in the Basidiomycota as well. Indeed, deletion and overexpression of the *cre1* gene in the *P. ostreatus* showed that *cre1*-mediated regulation of CAZyme activities is substrate-dependent (14). A recent study based on RNA interference (RNAi) silencing showed that the creA ortholog in *G. lucidum* can regulate cellulase enzyme activity and transcription of related genes (11). However, despite these studies, the role of the *cre1* gene in global gene regulation and its regulon in mushroom-forming fungi are unclear, hampering in-depth studies on the regulation of plant biomass degradation in the Basidiomycota.

Here, we investigated the effects of *cre1* gene deletion on the phenotype and global gene expression regulation in Coprinopsis cinerea. *C. cinerea*, also known as “Inky-cap mushroom'' forms complex fruiting bodies that autolyze to produce a black mass of spores. It is a widely used model in mushroom developmental biology (19, 20). At the same time, *C. cinerea* is a litter decomposer species, which is adapted to decomposing lignocellulosic substrates such as manure, hay, or straw. Litter decomposers represent an ecologically successful guild among mushroom-forming fungi, mostly in the Agaricales (21, 22). Unlike white and brown rot fungi which decompose wood, litter decomposers obtain carbon from nonwoody plant biomass. In *C. cinerea*, the availability of a self-fertile homokaryotic strain (AmutBmut *pab1-1*), along with widely used selection markers, including auxotrophic marker genes, facilitate genetic studies (19, 23, 24).

Genomic integration of foreign DNA occurs either by nonhomologous end joining (NHEJ) or homologous recombination (HR). NHEJ is widespread in fungi, while HR is very limited (25, 26), especially in the mushroom-forming fungi (27, 28), which complicates HR-dependent targeted gene deletion. The ratio of HR-mutants has been increased by disruption of the NHEJ genes *ku70*, *ku80*, or *lig4* in mushroom-forming fungi (26, 29–31). Another approach for preferentially utilizing HR-dependent gene integration is the split-marker gene deletion approach, which uses two overlapping fragments of the dominant marker with flanking target gene fragments (32). In Cordyceps militaris, the split-marker approach results in increased gene deletion compared to a single fragment (33). This approach is commonly used for genetic manipulations in filamentous fungi (34), but not in *C. cinerea*. Recently, genome editing using CRISPR/CRISPR-associated protein 9 (Cas9) has become popular in various fungi, including Agaricomycetes (35–42), and involves a report of using the split-marker together with the preassembled Cas9 ribonucleoproteins (Cas9 RNPs) in *P. ostreatus* (43).

In this study, we identified the *C. cinerea cre1* gene and generated two independent *C. cinerea cre1* mutants using preassembled Cas9 RNPs along with a PCR-based split-marker DNA repair cassette. Transcriptional changes were analyzed in the two *C. cinerea cre1* mutants using Quant-Seq. Our analysis highlights putative target genes of Cre1 in a litter decomposer basidiomycete. We detected differential expression of several carbohydrate metabolism genes, carbohydrate-active enzyme encoding genes (CAZymes) related to plant cell wall degradation, plasma membrane transporters, and several transcription factors. In addition, this work also provides a simple, efficient, and rapid approach for gene deletion in the mushroom *C. cinerea*.

## RESULTS

### Identification of the *C. cinerea cre1* gene and phylogenetic analysis.

**FIG 1:**
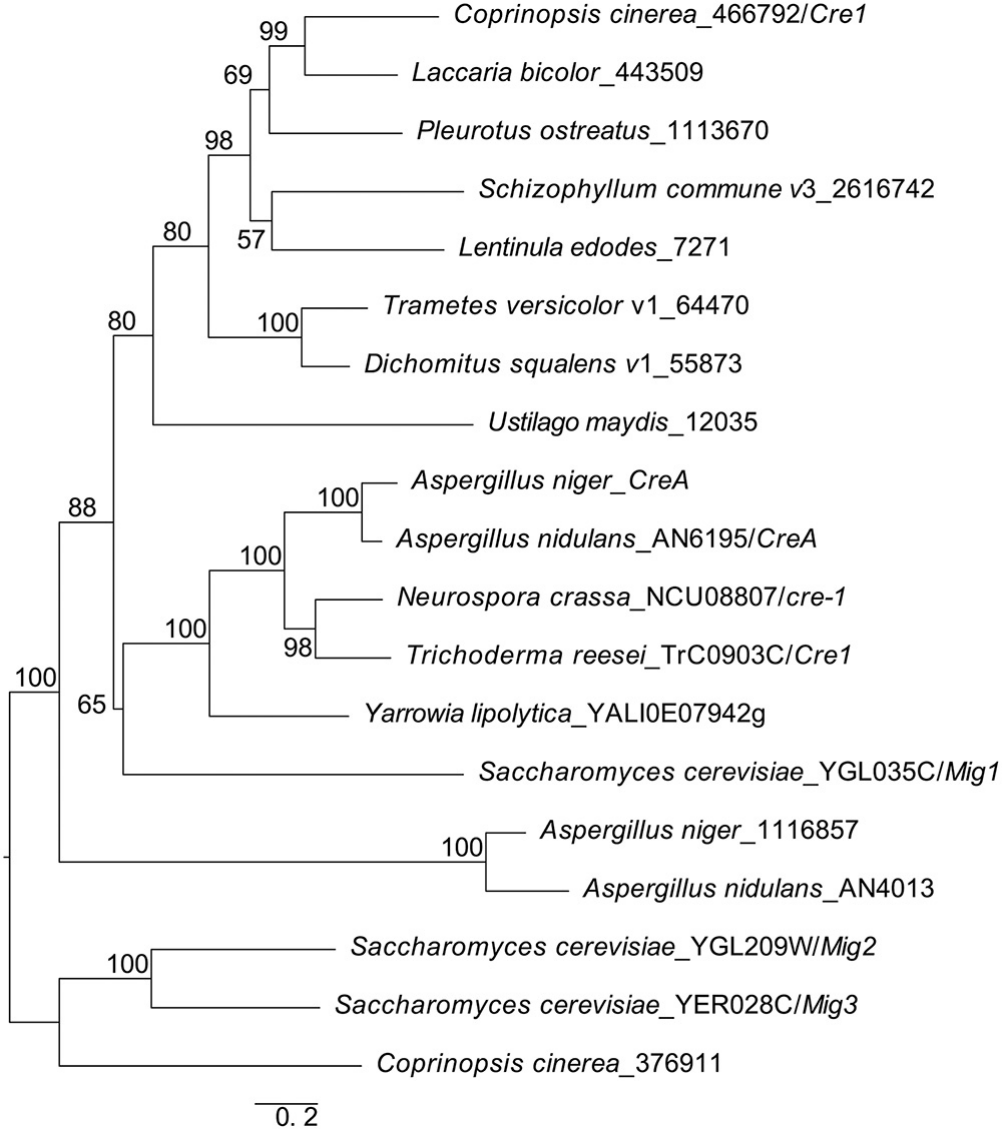
Maximum likelihood phylogenetic tree of C_2_H_2_ zinc finger transcription factors of selected filamentous fungi and yeasts. The tree reveals the monophyly of *C. cinerea* Cre1 (466792/Cre1) with experimentally characterized members of the family, including S. cerevisiae Mig1, A. niger CreA, A. nidulans CreA, N. crassa Cre-1, and of *T. reesei* Cre1. The numbers next to branches represent the bootstrap support values. JGI protein IDs are given for each protein used in the analysis.

We identified the Cre1 ortholog for *C. cinerea* using a reciprocal best hit strategy in protein BLAST and confirmed orthology using maximum likelihood analysis of the Cre1 orthologs and related C_2_H_2_ transcription factors (Fig. 1). The phylogeny strongly supported the monophyly of protein 466792 of *C. cinerea* with experimentally characterized members of the family, including Mig1 of S. cerevisiae, CreA of Aspergillus niger and A. nidulans, Cre-1 of Neurospora crassa, and Cre1 of Trichoderma reesei. On the other hand, sequences representing Mig2 and Mig3 of S. cerevisiae and closely related proteins of A. niger and A. nidulans were clustered separately. Based on these results, we designate this protein of *C. cinerea* as Cre1.

### Preassembled Cas9 RNPs together with a split-marker DNA repair cassette increases the number of transformants.

*C. cinerea* protoplast transformation using preassembled Cas9 RNPs together with a split-marker DNA repair cassette was compared with the split-marker DNA repair cassette alone method. We observed that the number of transformed colonies increased when RNPs were added together with the split-marker cassette compared with the split-marker DNA repair cassette alone transformation. Transformation with the split-marker DNA cassette alone yielded 78 colonies, whereas RNPs together with the split-marker DNA cassette in the same experiment yield 523 colonies within 5 days after transformation. Later, 96 colonies (48 from each method) were screened for deletion of the *C. cinerea cre1*.

### Identification of the *C. cinerea cre1* gene deletion strains.

**FIG 2:**
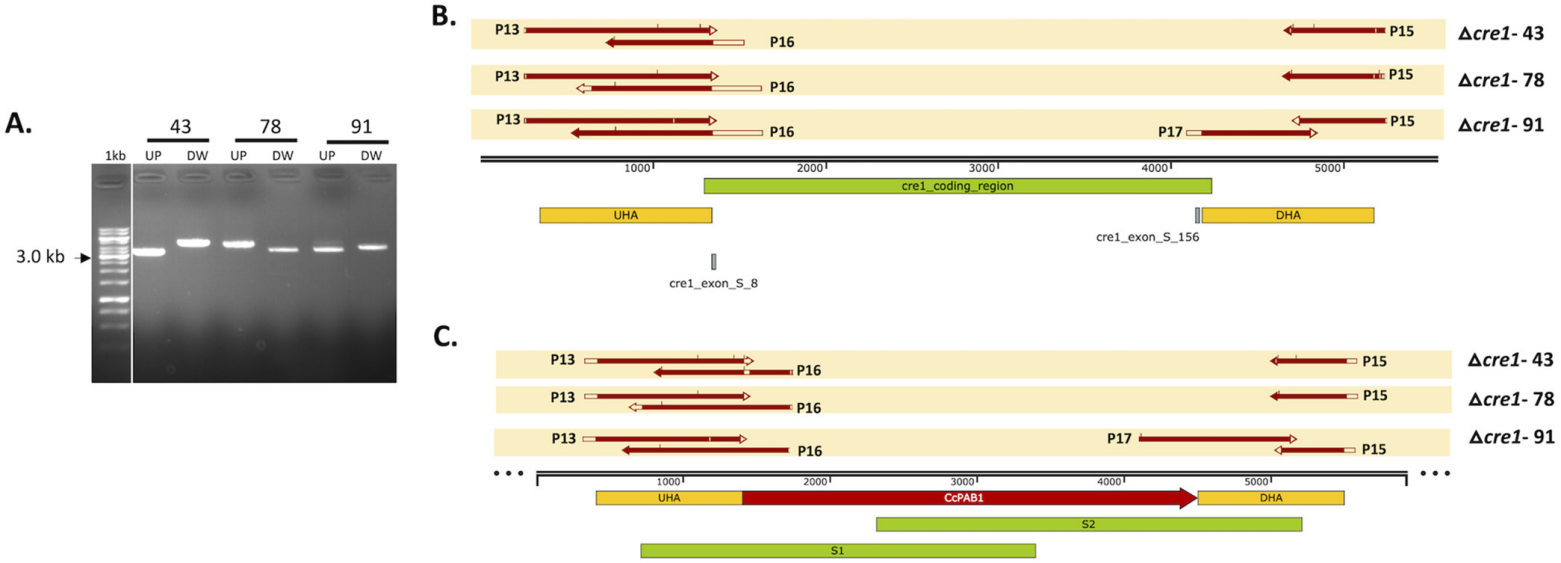
Confirmation of the *C. cinerea cre1* mutants by PCR amplification and DNA sequencing of the *pab1* marker gene along with the flanking region. PCR amplification of the upstream (UP) and downstream (DW) DNA fragments from the genomic DNA of the selected strains (43, 78, and 91) using specific primers (A). The reads from DNA sequencing of UP and DW PCR amplified fragments were aligned with the *C. cinerea cre1* gene locus (B) and the PABA transformation cassette (C). UP and DW are the DNA fragments used for DNA sequencing with primers P13, P16, P17 and P18, as described in the experimental procedures. UHA, homology arm; DHA, downstream homology arm; CcPAB1, *pab1* selection marker gene, cre1_coding_region, *C. cinerea cre1* gene locus; cre1_exon_S_8 and cre1_exon_S_156, positions of sgRNA binding.

We used the three PCR screening methods described in the experimental procedures to identify deletion strains among the putative transformants (Fig. S1). Using screening method 1, replacement of the target gene by the *pab1* marker cassette can be detected by the difference in the size of the PCR amplicon compared to wild type fungal strain (Fig. S2A and B). Screening method 2 is expected to show that true *cre1* deletion strains do not amplify the native *C. cinerea cre1* locus (Fig. S2C and D). Considering the above two screening methods, three strains, 43, 78, and 91, were selected for confirmation by DNA sequencing. All three strains were generated using the split-marker DNA repair cassette together with Cas9 RNPs. Final confirmation of the three deletion mutants by screening method 3 was performed by sequencing the upstream (UP) and downstream (DW) fragments as explained in the experimental procedures. The UP and DW DNA fragments amplified from all three deletion strains and their DNA sequencing results were aligned with the *C. cinerea cre1* gene locus or the para-aminobenzoic acid (PABA) transformation cassette are shown in Fig. 2A to C.

The results of DNA sequencing of the UP fragment using the P13 primer for all three deletion strains Δ*cre1*-43, Δ*cre1*-78, and Δ*cre1*-91 were aligned to the upstream homology arm (UHA). It shows that the UP fragment amplified (Fig. 2A) for all strains is from the *C. cinerea cre1* gene locus. Similarly, DNA sequencing reads of the UP fragment with the P16 primer aligned to the PABA gene but not to the *C. cinerea cre1* gene. This means that the PABA transformation cassette replaced the *C. cinerea cre1* gene locus in all three transformants. Similarly, the results of DNA sequencing of the DW fragment with the P15 primer aligned to downstream homology arm (DHA), confirming the position of the DW fragment at the *C. cinerea cre1* gene locus for all three deletion strains. DNA sequence data obtained with the P17 primer aligned to the PABA gene in the Δ*cre1*-91 deletion strain, but good sequencing results could not be obtained for the Δ*cre1*-43 and Δ*cre1*-78 mutants (Fig. 2B and C). DNA sequencing of the UP and DW fragments amplified from the genomic DNA of the mutants and their alignment to the *cre1* PABA transformation cassette showed integration of the PABA gene into the *C. cinerea cre1* locus (Fig. 2B and C). Our results show that all three selected strains (Δ*cre1*-43, Δ*cre1*-78, and Δ*cre1*-91) can be considered *C. cinerea* mutants. Only mutants Δ*cre1*-43 and Δ*cre1*-91 were used for further analysis.

Although we performed very stringent PCR screening for the identification of the clean mutants, it was later found that the genomic DNA of both mutants gave PCR amplification of the small *C. cinerea cre1* gene fragment at a high number of PCR cycles using two gene internal primers (P18 and P19). Even after oidiation of the mutant strains, the PCR DNA band persisted. We hypothesized this was because of variation in the recombination event of the PABA split-marker cassette and due to differences in functionality of the two RNPs, but this needs further investigation. Despite this uncertainty, we are confident that Δ*cre1*-43 and Δ*cre1*-91 represent *C. cinerea cre1* gene deletion mutants, which is confirmed by the results of Quant-Seq analysis (see below).

### *C. cinerea cre1* knockout slightly affects vegetative growth but not fruiting body formation.

**FIG 3:**
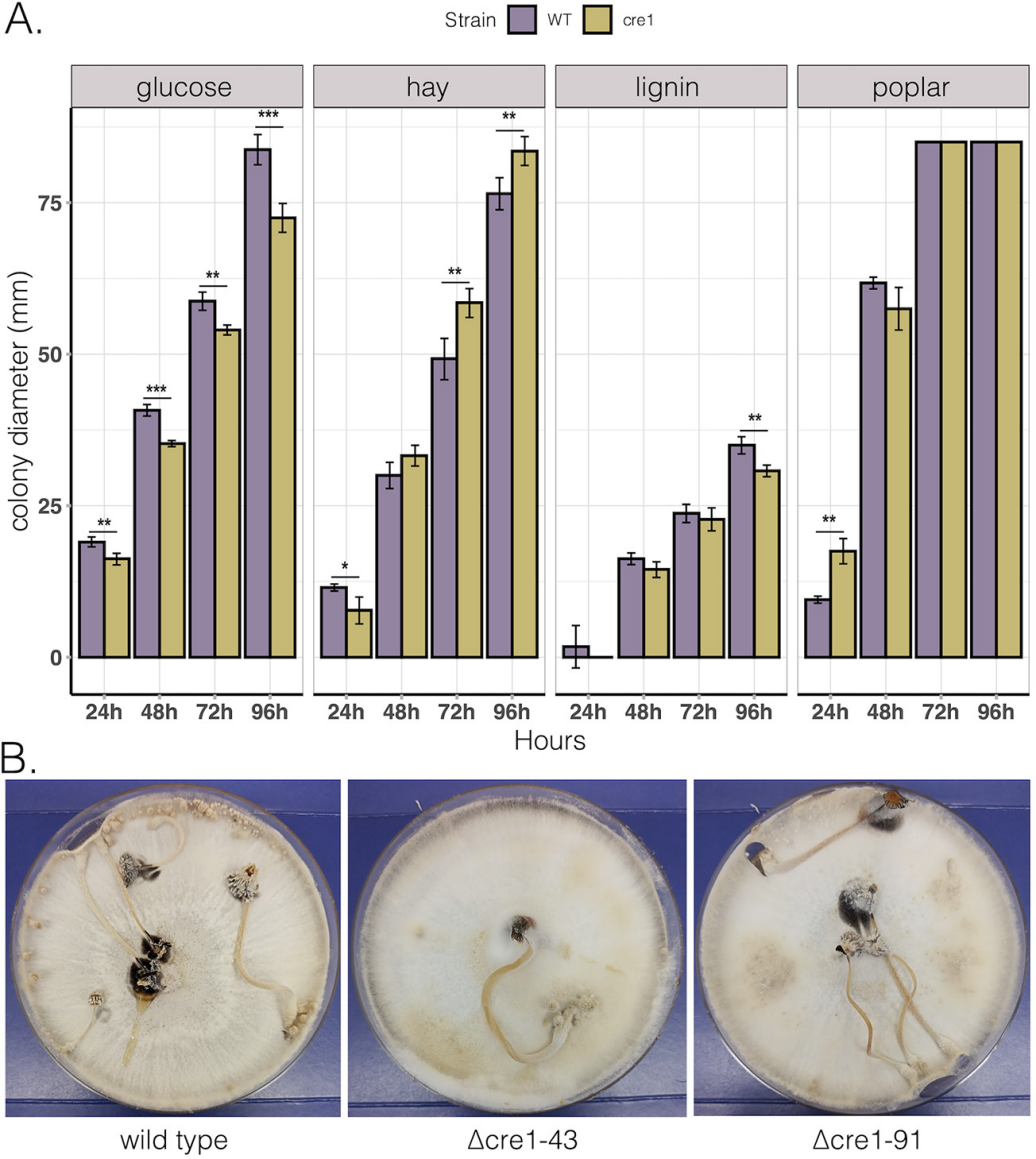
Phenotyping of wild type and *C. cinerea cre1* mutants. (A) Growth rate, as measured by colony diameter on media containing glucose and lignocellulosic plant biomass. By 72 h on poplar sawdust, the colonies reached the edge of the plates (albeit forming very loose, cobweb-like mycelia); therefore, bars are truncated on the plots. *, *P* < 0.05; **, *P* < 0.01; ***, *P* < 0.001. (B) Development of fruiting bodies in Coprinopsis cinerea (AmutBmut *pab1*) wild-type strain and Δ*cre1*-43 and Δ*cre1*-91 deletion strains after 14 days on YMG medium.

We grew wild type and deletion strains on media containing glucose, hay, lignin, and poplar sawdust. Of these, *C. cinerea* grew well on glucose- and hay-containing media, whereas on lignin and poplar sawdust, the colonies were loose and cobweb-like, indicating that these are suboptimal conditions for the fungus. On glucose-, sawdust-, and lignin-containing media, the wild-type strain grew slightly faster, while on media containing hay, the Δ*cre1* strains had a slight advantage (Fig. 3A). As hay is closest to the natural substrate of *C. cinerea*, we think the faster growth of the Δ*cre1* strains on hay may be attributable to derepressed cellulase and hemicellulose-encoding genes allowing the fungus to utilize hay faster than the wild type, which first had to restructure gene expression.

Both deletion strains Δ*cre1*-43 and Δ*cre1*-91 developed normal fruiting bodies on yeast/malt extract glucose (YMG) medium like the wild-type strain; no change was observed (Fig. 3B).

### Carbohydrate metabolism is broadly affected in *cre1* mutants.

In the Quant-Seq data for the *C. cinerea cre1* mutant and wild-type samples, approximately 74 to 94% of the reads were uniquely mapped to the *C. cinerea* genome (Table S5). We analyzed the effects of *C. cinerea cre1* deletion on the transcriptome. The differentially expressed genes in both mutants are listed in Table S3 and S4. In the Δ*cre1*-43 and Δ*cre1*-91 mutants, 938 and 1,081 genes, respectively, were significantly differentially expressed (*P* ≤ 0.05, fold change (FC) > 2) compared to wild-type strains. The data show that 592 genes were common to both mutants. In the Δ*cre1*-43 mutant, 658 genes were upregulated and 280 genes were downregulated, whereas in the Δ*cre1*-91 mutant, 682 genes were upregulated and 399 genes were downregulated. Of these, 401 and 191 genes are commonly up- and downregulated in Δ*cre1*-43 and Δ*cre1*-91, respectively.

Significantly enriched gene ontology (GO) terms were identified for the differentially expressed genes separately for both mutants separately for the up- and downregulated genes. The enriched GO terms largely overlapped between the two mutants, indicating similar transcriptomic changes after deletion of *C. cinerea cre1* (Table S6). Common enriched terms in the upregulated genes of *C. cinerea cre1* mutants showed a clear signal for processes related to carbohydrate metabolism (e.g., carbohydrate/organic/primary metabolic process, carbohydrate/cellulose/polysaccharide binding, hydrolase activity, and extracellular region) (Table S6). Interestingly, the upregulated genes of the *C. cinerea* Δ*cre1*-91 mutant were also enriched in terms related to transcription initiation and protein-DNA complex, which was not the case for the Δ*cre1*-43 mutant. GO terms related to arabinose metabolism and oxidoreductase activity were enriched only in the Δ*cre1*-43 mutant. Among the downregulated genes, oxidoreductase activity, iron binding, transmembrane transporter activity, extracellular space, and membrane-related terms were commonly enriched in both mutants. GO terms of interest related to G protein-coupled receptors (GPCR) can be observed in both mutants, whereas terms related to amino acid metabolism are mainly enriched in the Δ*cre1*-91 mutant (Table S6).

### Plant cell wall degrading CAZyme genes are broadly upregulated in *cre1* mutants.

We analyzed the differentially expressed genes of Δ*cre1*-43 and Δ*cre1*-91 mutants for the presence of genes encoding CAZymes. Of the 485 CAZymes in *C. cinerea*, we found that 101 and 102 were differentially expressed in the Δ*cre1*-43 and Δ*cre1*-91 mutant strains, respectively. Of these, 80 CAZymes were upregulated in each of the strains and 69 were common to both. In addition, 21 and 22 were downregulated in the Δ*cre1*-43 and Δ*cre1*-91 mutants, respectively, of which 13 were common. We classified CAZymes by their main substrate into groups acting on plant cell wall-degrading (PCWDE) and fungal cell wall (FCW) CAZymes according to Sahu et al. (44).

Among the differentially expressed genes in the *C. cinerea cre1* mutants, 82 genes were related to PCWDEs, 19 genes to FCW, and 20 genes to neither PCWDE nor FCW. Overall, 35.49% (82/231) PCWDE genes, 17.92% FCW genes (19/106), and 12.82% (20/156) non-PCWDE and non-FCW genes were differentially expressed in the *C. cinerea cre1* mutants. This indicates that PWCDEs are enriched among the differentially expressed CAZymes.

**FIG 4:**
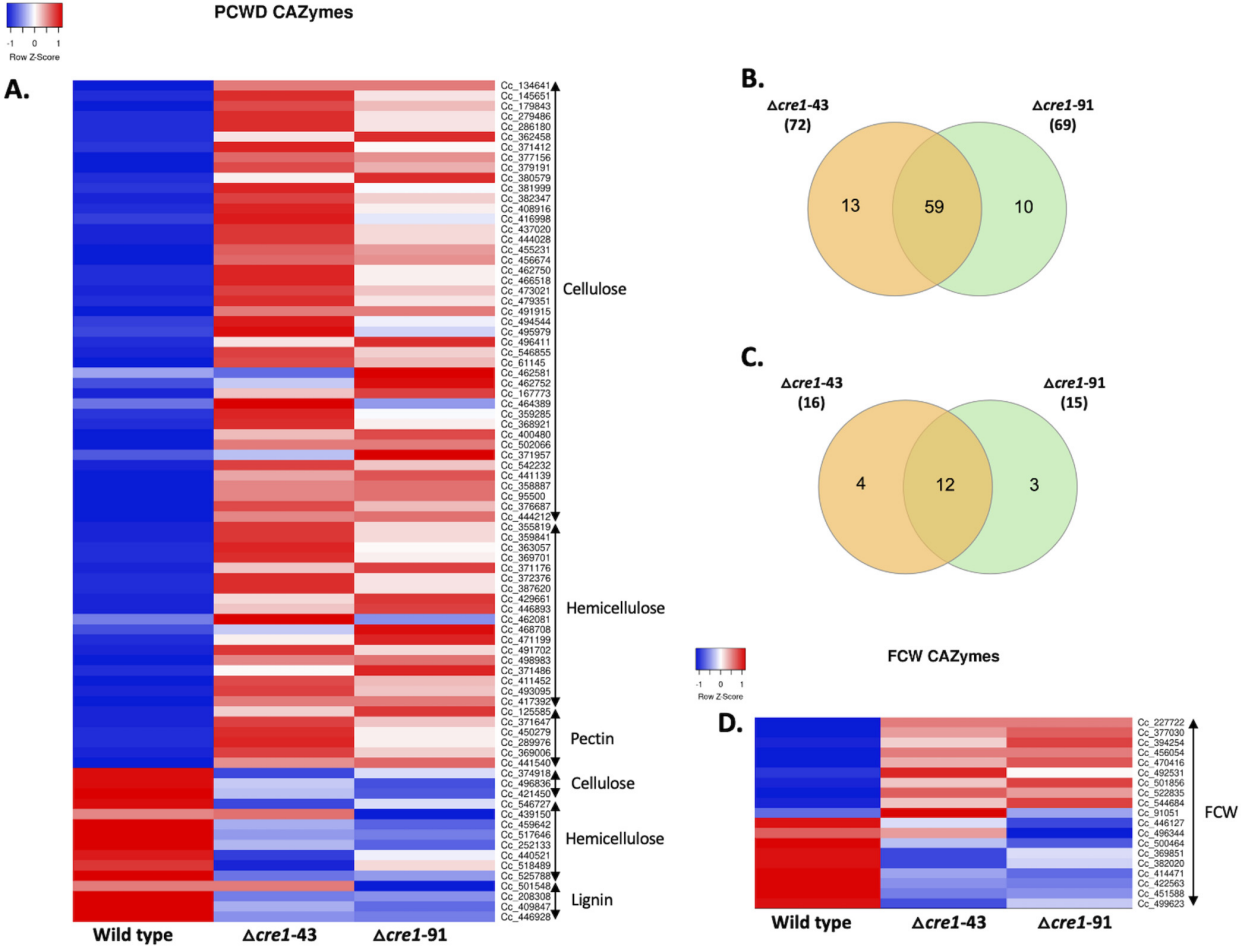
Differentially expressed CAZyme genes in the *C. cinerea* Δ*cre1*-43 and Δ*cre1*-91 mutants compared with the wild-type strain. (A) Heatmap showing the average CPM of differentially expressed plant cell wall degrading enzymes (PCWDEs) genes (JGI protein IDs) according to their main substrate of action (cellulose, hemicellulose, ppectin,and lignin) for the wild type, Δ*cre1*-43, and Δ*cre1*-91 mutants. (B and C) Venn diagram showing the common significantly differentially expressed PCWDE and FCW genes between the Δ*cre1*-43 and Δ*cre1*-91 mutants, respectively. (D) Heatmap showing the average CPM for the differentially expressed fungal cell wall (FCW)-related CAZyme genes (JGI protein IDs) for the wild type and Δ*cre1*-43 and Δ*cre1*-91 mutants.

We found a total of 67 upregulated PCWDE genes, of which 53 genes are commonly upregulated in both mutants (Fig. 4A; Table S7). In terms of predicted substrate, cellulase-encoding genes were dominant in both mutants, followed by hemicellulase and pectinase encoding genes (Fig. 4A). We found no gene predicted to be related to lignin degradation among the upregulated ones, though it should be noted that as a litter decomposer, *C. cinerea* possesses fewer lignin-related genes than white rot fungi (21). Fifteen PCWDE genes were downregulated (Fig. 4A and B; Table S7). These included four predicted lignin-modifying enzyme genes (LMEs), one class II peroxidase (AA2, protein ID: 446928) and three multicopper oxidases from the AA1 family (Table S7).

Among the differentially expressed cellulose-related CAZymes, the Auxiliary Activity Family 9 (AA9) family and CBM1 domain-containing genes were most abundant. Fifteen AA9 genes were differentially expressed, which are predicted to encode proteins with copper-dependent lytic polysaccharide monooxygenase (LPMOs) activity, and interestingly, all were upregulated in the *cre1* deletion strains except one. We also found 17 CBM1 domain-containing genes among upregulated ones (none among downregulated); most of these were predicted to have enzymatic activities and belong to the GH5, GH10, GH11, GH30, GH62, and GH131 families. Other differentially expressed genes were found in glycoside hydrolase (GH) families, including GH1, GH3, GH5_22, GH6, and GH7. Two expansins were upregulated in the mutants, but two were also downregulated. Expansins have been suggested to be active on both the plant and the fungal cell wall (45). Among the 10 upregulated FCW genes, nine were upregulated in both mutants, while out of the nine downregulated FCW genes, only three were significantly downregulated in both mutants (Fig. 4C and D; Table S7).

### Multiple transcriptional factors are affected by the *cre1* deletion.

**FIG 5:**
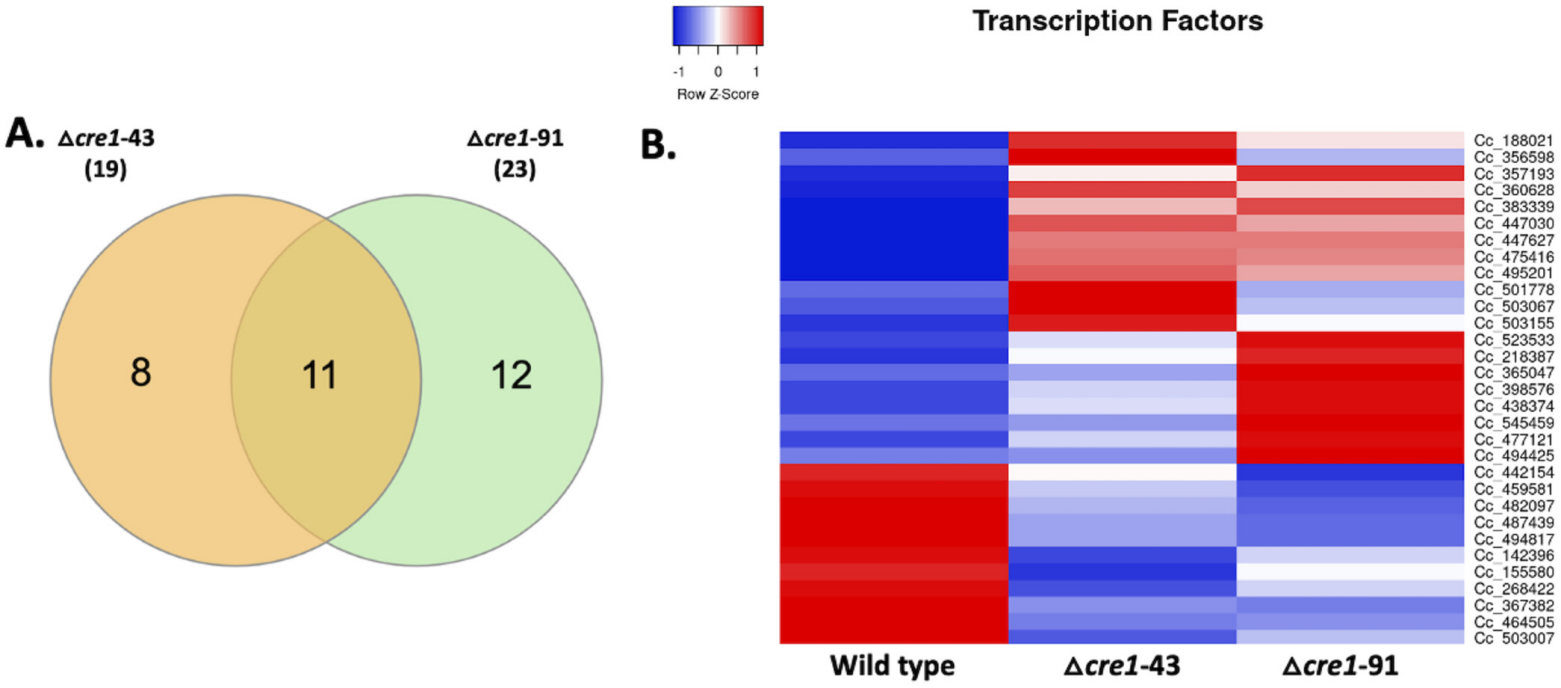
Differentially expressed transcription factor genes in the *C. cinerea* Δ*cre1*-43 and Δ*cre1*-91 mutants compared with the wild-type strain. (A) Venn diagram showing the common significantly deregulated genes between the Δ*cre1*-43 and Δ*cre1*-91 mutants. (B) Heatmap showing the average CPM of transcription factor genes for the wild-type strain and Δ*cre1*-43 and Δ*cre1*-91 mutants.

In total, we found 31 differentially expressed transcription factors in our Quant-Seq data of *C. cinerea cre1* mutants. Of the 31 transcriptional factors (TFs), 20 TFs were upregulated and 11 TFs were significantly downregulated in at least one of the mutants (Fig. 5B; Table S8). We identified 19 and 23 TFs significantly deregulated in Δ*cre1*-43 and Δ*cre1*-91, respectively. Eleven differentially expressed TFs were common in both mutants, of which nine were upregulated and two were downregulated (Fig. 5A).

Genes encoding high-mobility-group box domain-containing proteins (HMG superfamily) were most abundant among the differentially expressed genes. Five TFs containing the HMG box domain were differentially expressed, of which three were upregulated and two were downregulated. We also found that the *C. cinerea* ortholog (protein ID: 447627) of the *S. commune roc1* transcription factor was significantly upregulated in both mutants. Roc1 was recently described as a regulator of cellulase genes in *S. commune* (46). Similarly, a heat shock factor-type gene (protein ID: 523533) related to the S. cerevisiae *skn7* transcription factor was upregulated in both mutants. In addition, the upregulation of an *Ecd* family gene (protein ID: 438374) that shares homology with the Schizosaccharomyces pombe *sgt1* and a DNA2/NAM7 helicase-like domain-containing transcription factor gene (protein ID: 447030) was observed in the data. The complete list of the transcription factors is given in Table S8.

### Major facilitator superfamily and oligonucleotide transporters are extensively deregulated.

**FIG 6:**
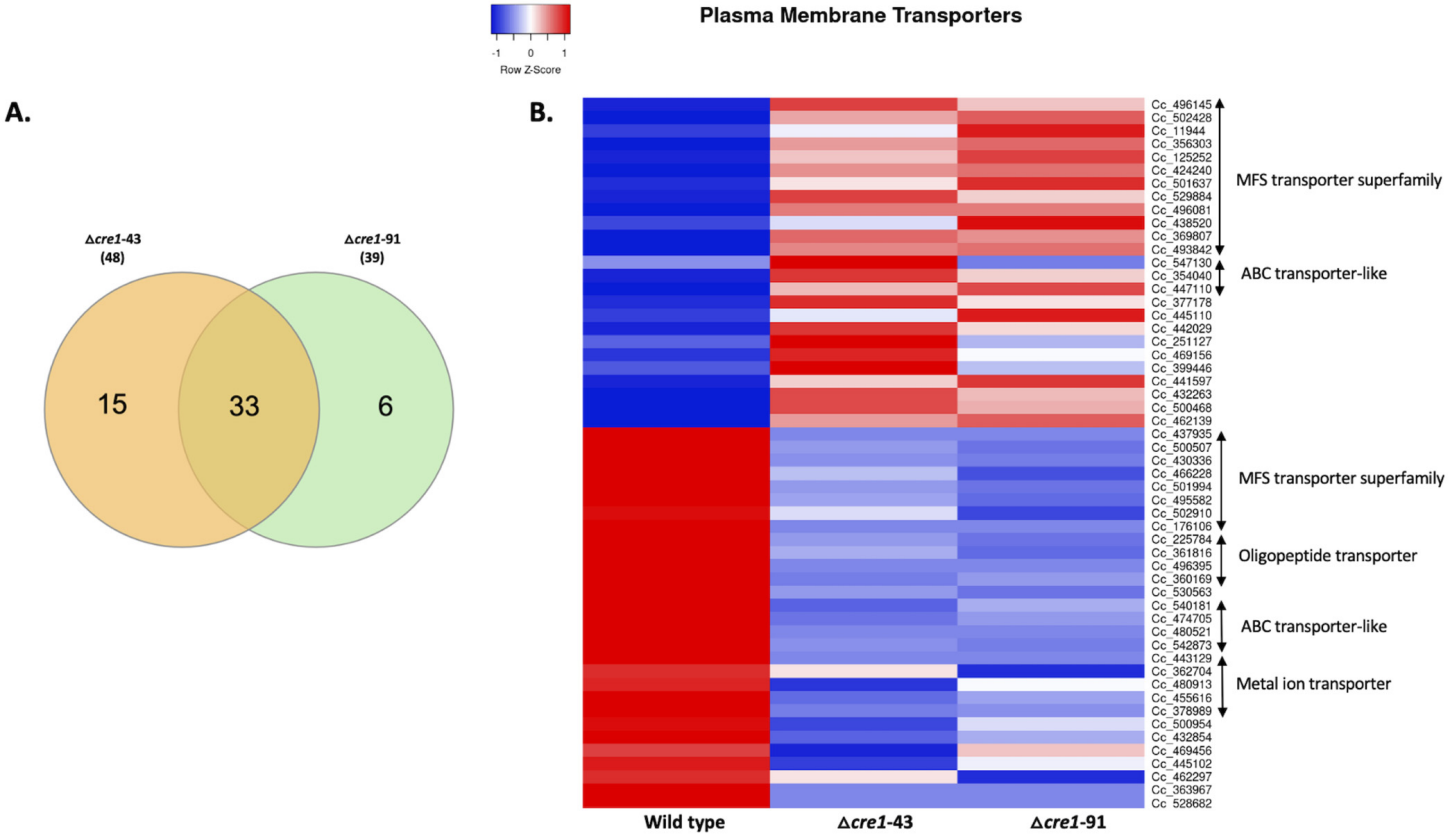
Differentially expressed plasma membrane transporter genes in the *C. cinerea* Δ*cre1*-43 and Δ*cre1*-91 mutants compared with the wild-type strain. (A) Venn diagram showing the common significantly affected plasma membrane transporter genes between Δ*cre1*-43 and Δ*cre1*-91 mutants. (B) Heatmap showing the mean CPM across replicates of differentially expressed plasma membrane transporter genes for wild type and Δ*cre1*-43 and Δ*cre1*-91 mutants.

In Quant-Seq data from *C. cinerea cre1* mutants, 54 genes encoding plasma membrane-associated transporter proteins showed a significant change in expression (Fig. 6A and B). Twenty-five and 29 of these genes were up- and downregulated, respectively (Table S9). The most frequently differentially expressed family among these genes was the major facilitator superfamily (20 genes).

In the Quant-Seq data, four genes containing InterPro domains related to the oligopeptide transporter superfamily were observed to be downregulated in *C. cinerea cre1* mutants. Several ion transporter-related proteins such as phosphate, nickel/cobalt, potassium, chromate, acetate, and zinc/iron permease genes were also downregulated (Table S9). In addition, several ABC transporter-like proteins, EamA domain, amino acid/polyamine transporter I, purine/cytosine permease, SH3-like domain superfamily, RTA-like protein, and STAS domain superfamily-related genes were also affected; the complete list is given in Table S9.

## DISCUSSION

In this study, we used a split-marker DNA repair cassette along with Cas9 RNPs to generate knockout strains of the carbon catabolite repressor *cre1* in the model mushroom C. cinerea and then assessed the transcriptional changes in the mutant strains. *C. cinerea* is a widely used model mushroom to study various molecular aspects of mushroom biology, including fruiting body development, sexual development, and sporulation (19, 20, 47–50). *C. cinerea* is also an efficient degrader of lignocellulosic plant biomass (51, 52). It is a litter decomposer fungus adapted to degrade herbaceous plant biomass (21).

Genetic transformation protocols exist for the transformation and gene deletion of *C. cinerea* (39, 53). The laborious cloning of plasmid-based DNA repair cassettes and low HR-mediated genomic integrations is a major obstacle to performing high-throughput gene deletion studies in *C. cinerea*. Gene deletion via split-marker DNA cassettes is commonly used for genetic studies in yeasts and filamentous fungi such as Fusarium oxysporum (32, 54), however, it has not yet been applied to *C. cinerea*. Here, we used a split-marker DNA transformation approach with or without *in vitro* assembled Cas9 RNPs. The results show that Cas9 RNPs with split-marker DNA repair cassettes increased the number of colonies more than six times compared to that without Cas9 RNPs. No *cre1* deletion strain was found among the examined transformants generated using the split-maker DNA cassette without Cas9 RNPs. The increase in the number of transformants and the identification of *C. cinerea cre1* deletion strains by using the split-marker DNA cassette with the Cas9 RNPs approach suggests that Cas9-mediated target-specific nicking may increase site-specific homologous recombination, which ultimately contributes to the integration of foreign DNA into the genome. Both Δ*cre1*-43 and Δ*cre1*-91 mutants showed integration of the *pab1* marker cassette into the *C. cinerea cre1* gene locus, demonstrating that the split-marker approach along with Cas9 RNPs is efficient for targeted gene deletion in *C. cinerea*.

On glucose-, sawdust-, and lignin-containing media, the wild-type strain grew slightly faster, while on media containing hay, the Δ*cre1* strains had a slight advantage (Fig. 3A). As hay is closest to the natural substrate of *C. cinerea*, we think the faster growth of the Δ*cre1* strains on hay may be attributable to derepressed cellulase and hemicellulose-encoding genes allowing the fungus to utilize hay faster than the wild type, which first had to restructure gene expression. Fruiting body formation is not significantly impaired in *C. cinerea cre1* deleted strains compared to wild-type strains. Further studies are needed to understand the role of *C. cinerea cre1* in fruiting body formation on different carbon sources.

We identified 658 to 682 upregulated and 280 to 399 downregulated genes in the two *cre1* mutants of *C. cinerea*; these showed a large overlap between the two independent mutants. It is important to note that these probably include genes both under direct and indirect control of Cre1 and assays of direct promoter binding (e.g., ChIP-Seq) will be necessary to distinguish between direct and indirect regulation. Nevertheless, the presence of both up- and downregulated genes suggests that Cre1 can act both as a repressor and an activator, respectively, consistent with previous reports (55–57). Differentially expressed genes also provide information on the potential regulon size of Cre1 in *C. cinerea*, which seems to be comparable to that reported in other species. In *T. reesei*, 815 and 697 genes were modulated by CRE1 in cellulose and glucose, respectively, and in Δ*cre1 T. reesei*, 905 genes were upregulated or downregulated in at least one of the conditions (56). For A. niger Δ*creA* mutant, 1,423 and 3,077 genes, around 12% and 26% of the total encoding genes, were differentially expressed on wheat bran and sugar beet pulp, respectively, at the early time point (57). In *Dichomitus squalens*, around 7% (1,042/15,295) of genes were repressed in glucose-containing medium (16).

The upregulated genes of *C. cinerea cre1* mutants were enriched for GO terms related to carbohydrate metabolism (Table S6), which was also evident in the analysis of PCWDE gene expression (Fig. 4). The upregulation of carbohydrate metabolism-related genes in *cre1* mutants is a phenotypic confirmation of the deletion of the *cre1* gene in *C. cinerea*. The role of the *cre1* gene in carbon metabolism, particularly in CCR, is already well-known for ascomycete fungi, but very little information is available for basidiomycetes (2, 6–8). Among the downregulated genes, it is interesting to see genes related to GPCR signaling; further studies can be performed to understand this. Previously, in A. nidulans it was suggested that trimeric G-proteins have a role in the CreA-independent regulation of CCR (10). The downregulation of genes with G protein-related GO terms in the mutants suggests that the *C. cinerea cre1* gene may be related to the normal expression of GPCR proteins in basidiomycetes. Oxidoreductase activity is also enriched in the downregulated genes of *C. cinerea cre1* mutants. Oxidoreductase activity might be related to auxiliary redox enzymes, which include LMEs (58–59). The GO terms affected in *cre1* gene deletion in various Ascomycota species are mainly carbohydrate metabolism, hydrolase activity, transporter activity, oxidoreductase activity, and membrane and transcriptional regulation (56–57), and similar GO terms were enriched in the *C. cinerea cre1* mutants as well. This indicates that the functional role of the *cre1* gene is conserved across phyla.

Our results indicated a broad upregulation of PCWDE-encoding CAZyme genes in the deletion strains even in the presence of glucose, suggesting that they are repressed either directly or indirectly by *C. cinerea* Cre1. This is consistent with the behavior of Cre1 orthologs in other species (2, 13, 14, 57), that is, repression of PCWDEs when the preferred carbon source, such as glucose, is present. In *P. ostreatus*, overexpression of *cre1* resulted in an increase in LMEs and a decrease in cellulose-degrading enzymes, whereas *cre1* knockout strains had a lower number of LMEs and slightly more carbohydrate-degrading enzymes (14). Our results also showed that cellulose-degrading enzyme genes were mostly upregulated, whereas LMEs are downregulated in *C. cinerea cre1* mutants. In the white rot basidiomycete *D. squalens*, glucose was reported to repress genes related to polysaccharide-degrading CAZymes and carbon catabolic genes (16). Similarly, glucose-induced reduction of cellulolytic enzyme expression was also shown in *P. ostreatus* (15). In our analysis, we show that deletion of *C. cinerea cre1* leads to upregulation of PCWDE CAZymes even in the presence of glucose. This suggests that the *C. cinerea cre1* gene is the main regulator of PCWDE expression during glucose-mediated CCR in the basidiomycete *C. cinerea*. Broadly, it can be concluded that *cre1*-mediated regulation of CAZymes in *C. cinerea* basidiomycetes is similar to that in ascomycetes.

Though, as a litter decomposer, *C. cinerea* has a somewhat reduced set of lignin-degrading enzyme genes compared to white rot species (e.g., *P. ostreatus*) (21); these genes were mostly downregulated in the *cre1* mutants. This suggests that genes encoding LMEs fall under a different regulation than cellulases, hemicellulases, and pectinases and is consistent with a recent analysis of the secretome of Cre1-deletion and overexpression strains in *P. ostreatus* (14). The regulation of LMEs in lignocellulose-degrading basidiomycetes thus appears to be different from that of cellulose/hemicellulose and pectin related genes and an interesting research avenue.

CreA in ascomycetes is known to exert its repressive effect on PCWDEs both directly and indirectly, through regulating and interacting with other transcription factors (13, 60). These transcription factors include ones specifically responsible for the activation of substrate-specific sets of enzyme-encoding PCWDEs, such as xylanases, cellulases, and pectinases (61–63). While several TFs regulating specific PCWDE genes have been described in ascomycetes, only a single gene, Roc1 of *S. commune* has been associated with cellulose degradation in the Basidiomycota (46). Our data revealed several transcription factors that are differentially expressed in *C. cinerea cre1* mutants, providing candidates for novel TFs related to plant biomass utilization.

The *C. cinerea* ortholog of Roc1 is upregulated in both independent *C. cinerea cre1* mutants. The *S. commune* transcription factor gene *roc1*, which is conserved among Agaricomycetes, was strongly upregulated in the cellulose-containing medium, and knockouts showed reduced growth on the cellulose medium, demonstrating its importance in regulating cellulose-degrading genes (46). ChIP-Seq analysis of *S. commune* Roc1 identified promoters of genes involved in lignocellulose degradation, particularly LPMOs (46). The upregulation of the *C. cinerea* Roc1 ortholog in *C. cinerea cre1* mutants may reveal a conserved link between the two transcription factors in regulating lignocellulose degradation.

We found that five putative transcription factors belonging to the HMG superfamily are deregulated in the *C. cinerea cre1* mutants. The HMG superfamily is a large and diverse group of chromosome-binding proteins that are mainly involved in transcriptional regulation, chromatin remodeling, DNA repair, replication, and recombination by altering chromatin architecture (64–66). In S. cerevisiae, a gene containing an HMG1 box domain encodes the mitochondrial histone protein Abf2p, which can be phosphorylated by the cAMP-dependent protein kinase; a strain with a defective *abf2* allele has severe effects on the regulation of mitochondrial DNA content during glucose repression (67). Deregulation of the HMG box superfamily in *C. cinerea cre1* deletion strains suggests that their interaction may control widespread gene regulation during CCR. Moreover, we also found a *C. cinerea* (523533) gene, which is reciprocal best blast hit to N. crassa *rrg-2* and S. cerevisiae *skn7* and was upregulated in both mutants. *Rrg-2*/*skn7* is a general stress regulatory transcription factor that affects diverse processes (68–69), including endoplasmic reticulum (ER) stress related to lignocellulase secretion (70). Whether the upregulation of the *C. cinerea* ortholog of *rrg-2* is the direct consequence of the deletion of *cre1*, or that of ER stress caused by the upregulation of PCWDEs remains to be investigated; nevertheless, these observations raise the possibility that ER stress also arises during lignocellulase secretion in the basidiomycetes.

Another widely conserved transcription factor in the *Ecd* family that shares homology with the S. pombe *sgt1* gene is upregulated in the *C. cinerea* Δ*cre1-91* mutant. S. pombe *Sgt1* regulates carbohydrate and amino acid metabolism (71). Although this group of transcription factors is functionally poorly known, *C. cinerea cre1*-mediated regulation of the *C. cinerea* ortholog of *sgt1* may be another way of controlling carbohydrate metabolism. Interestingly, a DNA2/NAM7 helicase-like putative transcription factor is also upregulated in both independent *C. cinerea cre1* mutants. The DNA2/NAM7 helicase-like protein is involved in unwinding DNA or RNA and affects DNA replication and transcription (72–74). Truncated *CRE1* (CRE1-96) in *T. reesei* (Rut-C30 strain) has been reported to upregulate the expression of helicase-like transcription factors (75). This suggests that helicases can be repressed by Cre1 to effectively orchestrate CCR in basidiomycetes. How the upregulation of this gene is linked to the deletion of *cre1* needs further investigation; we speculate it may be an indirect effect, which is spurred by the need for intense transcription in the hyphae.

Previously, CCR was reported in many fungal species to regulate the expression of the major facilitator superfamily (MFS) transporter genes (16, 57). In *D. squalens*, sugar transmembrane transporter activity was enriched in genes repressed on Avicel supplemented with glucose compared to Avicel alone (16). In N. crassa, Cre-1 has been shown to regulate not only PCWDEs and other TFs, but also sugar transporter genes that mediate the uptake of decomposition intermediates (60). Indeed, GO enrichment in the *C. cinerea cre1* mutants suggested that several genes related to transport were affected. These mainly included genes belonging to the major facilitator superfamily. It is known that proteins containing major facilitator superfamily and sugar/inositol-like domains are mainly involved in the transport of simple sugars, oligosaccharides, drugs, amino acids, nucleosides, organic alcohols, and acids (76–77). The deregulation of several transporters in our data suggest that they may be under the control of *C. cinerea* Cre1 to regulate the transport of specific sugars, peptides, and various ions during CCR.

Finally, our results support that the use of preassembled Cas9 RNPs together with a split-marker DNA repair cassette is an efficient approach for gene deletion in *C. cinerea*. Our data demonstrate that *C. cinerea cre1* acts as the carbon catabolite repressor in this fungus, and its deletion affects the expression of genes related to carbon metabolism, CAZymes (especially PCWDEs) upregulation, sugar transporters, ion transporters, and several transcription factors. It also supports the notion that Cre1 achieves a tight regulation of CCR through combined repression of PCWDEs, transcription factors that positively regulate PCWDEs (e.g., Roc1), and membrane transporters that could import simple sugars that can induce the expression of PWCDEs (60). In the future, the specific targets of the *C. cinerea cre1* gene can be identified, and the role of various transcription factors and transporters can be studied in more detail to understand the CCR and carbon metabolism in wood-degrading fungi.

## MATERIALS AND METHODS

### Strain, culture conditions, media, buffers, and reagent preparation.

The homokaryotic strain C. cinerea AmutBmut *pab1-1* (FGSC 25122) was used for the study (23). The fungus was cultured on YMG medium at 37°C under continuous light for 6 to 7 days to obtain oidia for protoplast preparation (78). The compositions and preparations of YMG, minimal medium (MM), regeneration medium, top agar, MM buffer, Mannitol, Maleic acid CaCl_2_ (MMC) buffer, and polyethylene glycol (PEG)/CaCl_2_ followed recipes described by Dörnte et al. (53).

### Comparative genomics and phylogenetic analysis.

The Cre1 ortholog (JGI protein ID: 466792) was identified in *C. cinerea* AmutBmut based on reciprocal best blast hits using the Neurospora crassa Cre-1 (NCU08807) protein as the query. The nucleotide sequence was retrieved from MycoCosm maintained by JGI for C. cinerea AmutBmut https://mycocosm.jgi.doe.gov/Copci_AmutBmut1/Copci_AmutBmut1.home.html (47).

A maximum likelihood phylogenetic analysis was performed to confirm the orthology of Cre1 to experimentally characterized carbon catabolite repressors from Ascomycota. For this purpose, we selected orthologs represented by reciprocal blast hits for key Basidiomycota (C. cinerea, D. squalens, Laccaria bicolor, Lentinula edodes, P. ostreatus, Schizophyllum commune, Trametes versicolor, and Ustilago maydis) and Ascomycota (Aspergillus nidulans, Aspergillus niger, N. crassa, S. cerevisiae, Trichoderma reesei, and Yarrowia lipolytica). For rooting the tree, we chose sequences of related C_2_H_2_ transcription factors of S. cerevisiae and Aspergillus spp. Protein sequences were retrieved from the MycoCosm or Uniprot databases or from the Saccharomyces Genome Database (79), aligned with PRANK 170427 (80), and trimmed using the built-in ‘strict’ settings of trimAl (81). Accession numbers are provided in Fig. 1. The resulting multiple sequence alignment was analyzed using RAxML version 7.0.2 under the PROTGAMMAWAG model of evolution with 1,000 rapid bootstrap replicates to assess branch support. Trees were rooted and visualized in FigTree version 1.4.4 (82).

### Single guide RNAs (sgRNAs) design and *in vitro* assembled Cas9 RNPs preparation.

The trans-activating CRISPR RNA (tracrRNA) and crRNA were commercially synthesized for the *in vitro*-assembled RNPs (IDT, USA). sgRNA protospacers were designed with the sgRNACas9 software using the *C. cinerea cre1* gene sequence (protein ID: 466792) (83). Two sgRNAs (*cre1_exon_S_8* and *cre1_exon_S_156*) from the 5′ and 3′ ends of the *cre1* gene were selected, which had minimal off-target effects in the *C. cinerea* genome (Table S1). The position of the sgRNAs binding on the *C. cinerea cre1* gene locus is shown in Fig. S3A. Each synthesized crRNA and tracrRNA was annealed and mixed with the commercially available Alt-R S.p.Cas9 Nuclease V3 (IDT, USA) along with Cas9 buffer to obtain the *in vitro*-assembled RNP complexes according to the manufacturer's instructions. Briefly, for each *in vitro*-assembled RNP mixture, 12 μL of equimolar RNA duplexes were first formed by mixing 1.2 μL of crRNA with 1.2 μL of tracrRNA from the stocks of crRNA (100 μM) and tracrRNA (100 μM) together with 9.6 μL of duplex buffer (IDT) and then incubated at 95°C for 5 min and cooled for 2 min. Next, 12 μL RNA duplex, 0.5 μL Cas9 (10 μg/μL), 1.5 μL Cas9 working buffer, and 1.0 μL duplex buffer were mixed and incubated at 37°C for 15 min. Then, 15 μL of RNP mix was used for protoplast transformation. One RNP reaction for each *cre1_exon_S_8* and *cre1_exon_S_156* sgRNA was prepared and used along with the purified split marker DNA repair cassette for protoplast transformation.

### PCR based split-marker DNA repair cassette preparation.

Para-aminobenzoic acid (PABA) auxotrophy in the *C. cinerea* AmutBmut *pab1-1* strain can be used for the selection of the transformants with *pab1* gene as a selectable marker (84). The split-marker DNA cassettes for the *pab1* (PABA) marker gene along with a ~700-bp upstream homology arm (UHA) and downstream homology arm (DHA) of *C. cinerea* were prepared using the double-joint PCR method (DJ-PCR) (85). First, 1 kb upstream of the 5′ end (UHA) and 1 kb downstream of the 3′ end (DHA) was amplified with the primers (P1 and P2, and P3 and P4, respectively). The *pab1* cassette along with the promoter and terminator (CcPAB1, 3097 bp) was amplified with primers P5 and P6 from vector pMA412 and used as a selection marker for *C. cinerea* (AmutBmut *pab1-1*) (86, 87). P5 and P6 primers have overlapping overhangs with *C. cinerea cre1* UHA and DHA fragments. The vector map for the split marker cassettes (S1 and S2) along with primer locations can be found in Fig. S3B. Each purified 1-kb UHA or DHA fragment was fused to the 3-kb PABA cassette in separate tubes to generate a long common DNA fragment using the DJ-PCR protocol (UP-PABA or PABA-DOWN) (85) with Phusion Green Hot Start II high-fidelity PCR master mix (Thermo Scientific). Finally, the Split1 (S1) cassette was amplified from UP-PABA using P7 and P8. Similarly, Split 2 (S2) was amplified from the PABA-DOWN using P9 and P10. Both S1 and S2 have ~1,081-bp long overlapping *pab1* marker gene fragments, which are required for recombination between split DNA fragments. The protocol for generating a split-marker DNA repair cassette for gene deletion is described in more detail elsewhere (54, 85, 88). All primer positions are shown in Fig. S3A and B and their DNA sequences are given in Table S1. Primers for the DJ-PCR were designed using the Gibson assembly primer design function of SnapGene software (Insightful Science; https://www.snapgene.com/). Fig. S4 shows the purified S1 and S2 DNA cassettes used for protoplast transformation.

### PEG-mediated protoplast transformation using Cas9 RNPs and split-marker DNA cassette mix.

*In vitro* assembled Cas9 RNPs for both ends of the *C. cinerea cre1* gene were used together with 10 μg of the amplified split-marker DNA (S1 and S2) for PEG-mediated transformation of *C. cinerea* protoplasts. *C cinerea* protoplast transformation was done as per the protocol described by Dörnte et al. (53) with minor modifications. Briefly, 0.5× 10^8^ oidia were harvested and washed with MM buffer, then resuspended in 950 μL MM buffer and mixed with 50 μL Protoplast F enzyme (Megazyme, Ireland). The mixture was incubated for at least 2.5 to 3 h to generate ~70 to 90% protoplast in suspension. The protoplast suspension was washed with MMC buffer to stop the reaction and then resuspended in 200 μL of MMC buffer for two transformation reactions. Further, the protoplast solution was divided into two Falcon tubes, one for the split-marker DNA repair cassette alone transformation, and another for the RNPs together with the split-marker DNA repair cassette. To one tube, 25 μL PEG/CaCl_2_, 15 μL each RNP solution, and 10 μg of the split-marker DNA repair cassettes (S1 and S2) were added, and to another tube similar components except the RNP solution were added. The putative fungal transformants were picked from the minimal medium selection plates between 3 and 6 days of incubation at 37°C. Each colony was separately picked and placed on separate minimal medium plates. Genomic DNA was then isolated and selected fungal transformants were further analyzed using PCR.

### PCR screening of the transformed colonies for *C. cinerea cre1* gene deletion.

Colony PCR was performed using the crude genomic preparation protocol described previously (89). Three screening methods were used for PCR screening and confirmation by sequencing (Fig. S1). For screening method 1, primers P11 and P12 provided a size difference between the *C. cinerea cre1* wild-type allele (3.36 Kb) and the *pab1* cassette (3.62 Kb) in the putative transformants. All colonies were screened again using screening method 2, with one primer (P13) upstream of the UHA and another primer (P14) located within the *C. cinerea cre1* gene locus. We used 34 to 35 PCR cycles to identify the true deletion strain with complete removal of the *cre1* gene locus. All mixed and ectopic transformants should yield a PCR signal in screening method 2, but a true *cre1* gene deletion strain does not yield an amplicon.

For screening method 3, selected colonies were grown again on a YMG medium and then genomic DNA was isolated from the fungal mycelium using the CTAB method (90). PCR was used to amplify the large 3.0-kb upstream fragments (UP) using the external forward primer (P13) and the PABA internal primer (P8). Similarly, the PABA internal forward primer (P9) was used together with the external downstream reverse primer (P15) for amplification of the downstream fragment (DW). These amplified PCR fragments indicate the integration of the *pab1* marker into the *cre1* locus of *C. cinerea*. Finally, the UP and DW fragments were used for DNA sequencing with primers P13 and P16 (UP) and P15 and P17 (DW). All primer positions and DNA sequences are shown in Fig. S1 and S3 and Table S1. Later, two internal primers for the *C. cinerea cre1* gene (P18 and P19) were also used for PCR confirmation of mutants from genomic DNA (Fig. S3A).

### RNA isolation and sequencing.

RNA was isolated from the wild-type (C. cinerea AmutBmut *pab1-1*) strain (3 replicates) and the Δ*cre1*-43 and Δ*cre1*-91 mutants (2 replicates for each independent mutant). All colonies were grown on YMG medium (0.2% glucose) overlaid with cellophane for 84 h in the dark at 28°C. Mycelia were collected and homogenized in liquid nitrogen using a mortar and pestle. Total RNA was isolated using the Quick RNA minikit (Zymo Research, USA). Preparation of cDNA libraries for Quant-Seq, purification, and sequencing were performed by iBioScience Ltd. (Pécs, Hungary) on an Illumina NextSeq instrument to achieve a sequencing depth of at least 10 million reads per sample.

### Quant-Seq data analysis.

The reference genome sequence (C. cinerea Amut1Bmut1 pab1-1 v1.0) and gene annotation (Copci_AmutBmut1_GeneModels_FrozenGeneCatalog_20160912.fasta) were downloaded from the JGI database. Read quality was checked using FastQC (91). Adapter sequences, polyA reads, and low-quality tails were removed using bbduk version 38.92 (92). Trimmed reads were mapped to the reference genome using STAR version 2.6.1a_08-27 (93). Trimming and mapping parameters were set according to the manufacturer's recommendations (Lexogen, Austria). Only CDS gene annotations were considered in the read to gene assignment step with a 400-bp overhang added at the 3′ ends of each gene. For the assignment, the featureCounts Rsubread_2.6.4 (94) function of the Rsubread package was used. For differential gene expression analysis, the edgeR version 3.34.0 (95) and limma version 3.48.3 (96) packages were used. Genes with a fold change ≥ 2 and a Benjamini-Hochberg (BH) adjusted *P* value ≤ 0.05 were considered upregulated (deg_status = 1). Genes with a fold change ≤ −2 and a BH adjusted *P* value ≤ 0.05 were considered downregulated (deg_status = −1). We also generated the list of InterPro (IPR) terms, IPR descriptions, and GO terms associated with the *C. cinerea* protein ids based on the output of InterProScan version 86 (Table S2). We analyzed the Quant-Seq data of the Δ*cre1*-43 and Δ*cre1*-91 mutants independently to find differentially expressed genes compared with wild type samples; the data obtained are shown in Table S3 and Table S4, respectively. The normalized average count per million (avg CPM) reads for the mutants and wild type is shown in Table S5.

### Functional annotations.

GO enrichment analyses were performed for differentially expressed genes separately for the Δ*cre1*-43 and Δ*cre1*-91 mutants using the topGo R package (97). We used the classicFisher algorithm to identify GO terms with a *P* value ≤ 0.05. GO terms for all *C. cinerea* protein ids can be found in Table S2.

The putative CAZymes in *C. cinerea* were annotated along with their major substrate based on previous publications (51, 98–108) and used for identification of PCWDEs and FCW-related CAZymes in our data. Putative transcription factors were identified based on lists provided in the study by Krizsán et al. (107). Putative transporter proteins localized in the plasma membrane were taken from the list in the study by Sahu et al. (44). We also confirmed their subcellular localization with WoLF PSORT and used proteins with predicted plasma membrane localization for analysis (109). Expression heatmaps were generated using the online tool HeatMapper (110) and Venn diagrams were generated using InteractiVenn (111).

### Phenotyping and fruiting assays.

To compare the growth rate of the *C. cinerea cre1* deletion strain to that of the wild type on different carbon sources, small agar plugs were cut out from 3-day-old cultures of the wild type and the *C. cinerea* Δ*cre1*-43 strain and placed on minimal media supplemented with either 2% glucose, 2% minced hay, 2% lignin (Lignin, alkali, Sigma-Aldrich), or 2% poplar sawdust. The experiment was carried out in 4 replicates. Colony diameters were measured every 24 h until 96 h. Statistical analysis was performed using Student's *t* test in R.

For analysis of fruiting body development, *C. cinerea cre1* mutants and the wild-type strain (Coprinopsis cinerea AmutBmut *pab1-1*) were cultured on YMG for 5 days at 37°C in the dark and then transferred to a 12/12 h light/dark cycle at 28°C for 5 to 7 days to analyze fruiting body development.

### Data availability.

The Quant-Seq data used for the gene expression analysis have been deposited in NCBI’s Gene Expression Omnibus (112) and are accessible through the GEO series accession number GSE205749.
