# Supplementary material for: Preassembled Cas9 Ribonucleoprotein-Mediated Gene Deletion Identifies the Carbon Catabolite Repressor and Its Target Genes in Coprinopsis cinerea
Source: Appl Environ Microbiol. 2022 Nov 14;88(23):e00940-22. doi: 10.1128/aem.00940-22 (PMC9746306; doi:10.1128/aem.00940-22)
Supplement: Supplemental file 1 — Fig. S1 to S4 and legends for Tables S1 to S9. Download aem.00940-22-s0001.pdf, PDF file, 0.4 MB [file aem.00940-22-s0001.pdf]

Supporting Information for

Pareek et al

Pre-assembled Cas9 ribonucleoprotein-mediated gene deletion identifies the carbon catabolite repressor and its target genes in Coprinopsis cinerea

Applied and Environmental Microbiology 2022

Contents:

- Supplementary Figures 1-4
- Legends for supplementary tables

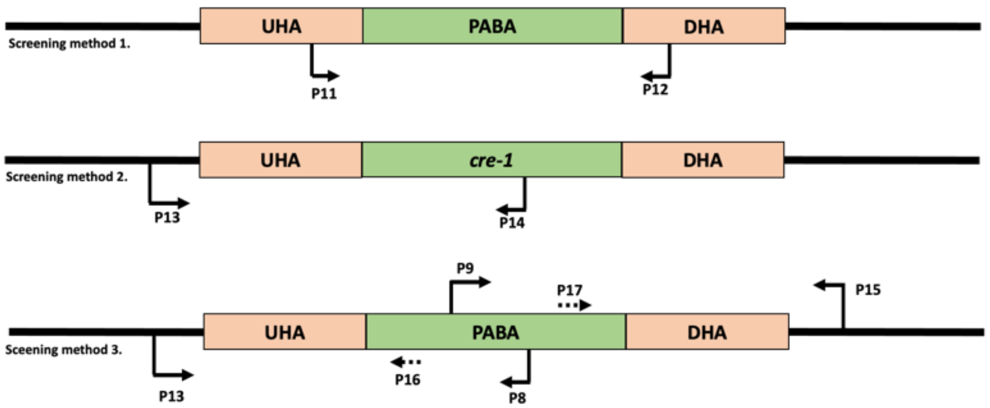

Fig. S1. Different screening methods were used to screen the putative *C. cinerea cre1* deleted strain. **Screening method 1**. transformants with PABA integration and wild-type gene PCR amplicons can be distinguished by size using the flanking diagnostic primers (P11 and P12). Here, the size of the wild-type *C. cinerea* cre1 gene locus = 3.36 KB; the size of the mutant = 3.62 (with PABA insertion). **Screening method 2**. true deletion mutants yield no amplification with the external forward primer P13 and the *cre1* gene internal reverse primer P14. **Screening method 3**. Provide information on the location of the deleted locus using external primer P13 and another PABA internal primer P8. Similarly, at the other end, use primers P9 and P15. P16 and P17 are used for DNA sequencing along with P13 and P15 for final confirmation of the amplified product.

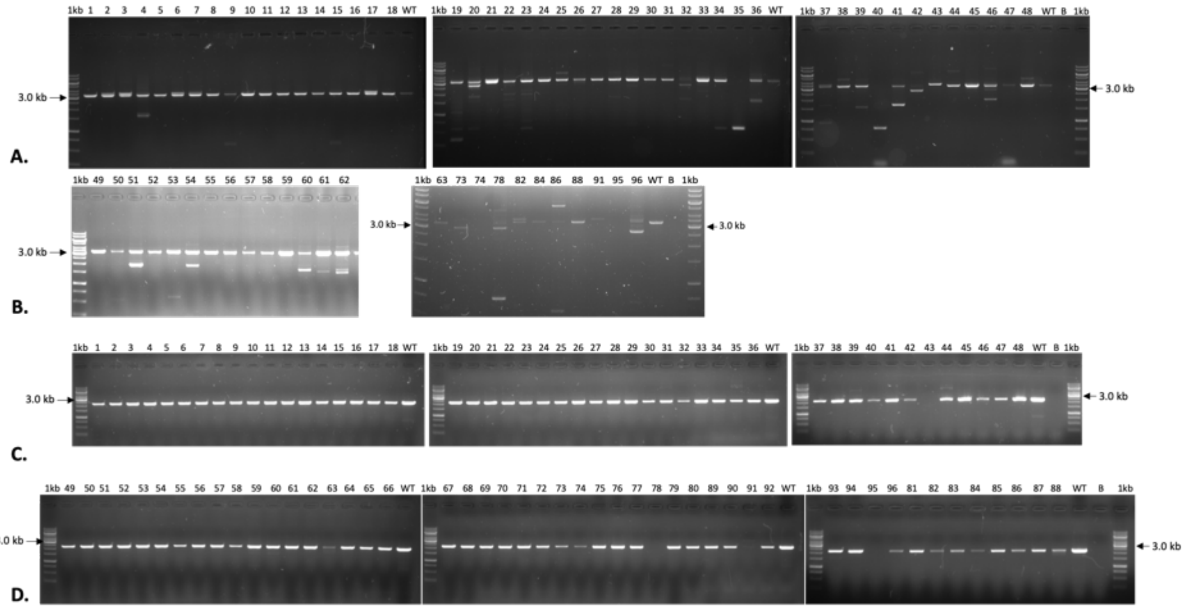

Fig. S2 (A-B). PCR screening of the putative *C. cinerea* cre1 mutants (1-96) using screening method 1 (P11 and P12 primers). Colony numbers 1-24; 49-72 were obtained by the transformation approach with split-marker DNA repair cassette alone. Colony numbers 25-48; 73-96 were transformed by the RNPs along with the split marker DNA

repair cassette. Not all samples are shown after transformant 63. (**C-D**). PCR screening of *C. cinerea cre1* mutants (1-96) using screening method 2 (P13, P14 primers). The loading order of the samples is discontinuous after transformant 80. WT - Wild type strain, B - blank/water control, 1kb- GeneRuler 1 kb DNA ladder.

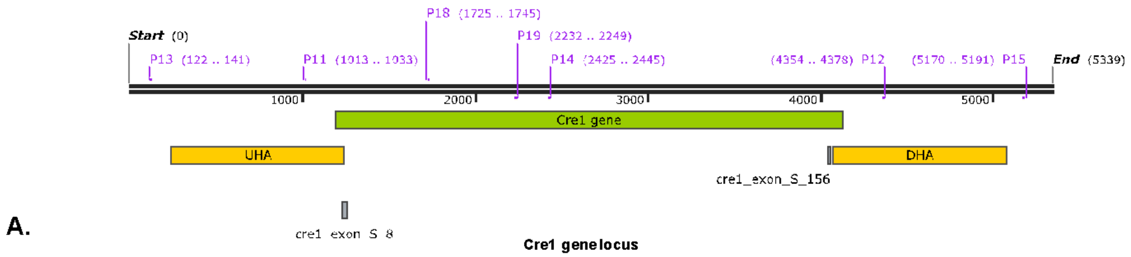

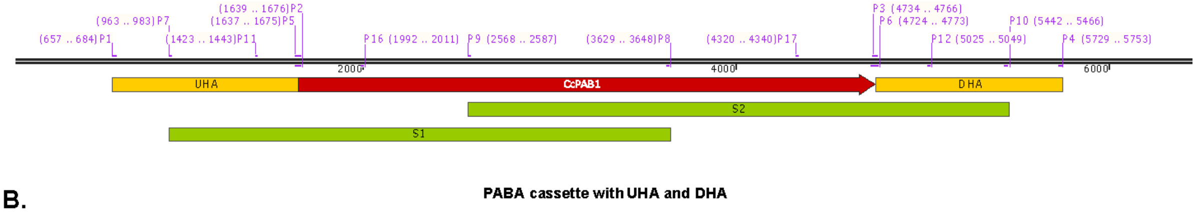

Fig. S3 (**A**) DNA map shows the *C. cinerea cre1* gene locus together with the upstream homology arm (UHA), the downstream homology arm (DHA) and the sgRNA binding sites (cre1_exon_S_8 and cre1_exon_S_156). P13, P11, P18, P19, P14, P12 and P15 are primers used for screening the transformants. (**B**) DNA map of *cre1* PABA cassette showing the position of the selection marker *pab1* gene (CcPAB1 or PABA) along with the upstream homology arm (UHA) and downstream homology arm (DHA). The positions of the split marker cassettes are also indicated as split 1 (S1) and split 2 (S2). The different primer positions used for split cassette preparation, screening, and sequencing of the mutants are shown here (P1, P7, P11, P5, P2, P16, P9, P8, P17, P3, P6, P12, P10, and P4).

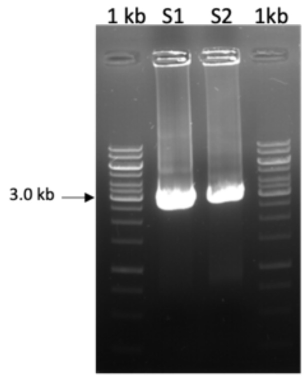

Fig. S4: PCR-amplified and purified split 1 (S1) and split 2 (S2) DNA repair cassette used for PEG -mediated protoplast transformation.

Table S1: DNA sequences of the sgRNAs protospacer and primers used in the study.

Table S2: *C. cinerea* protein IDs along with its InterPro accession number, InterPro description and Gene Ontology (GO) IDs.

Table S3: Quant-Seq data analysis of Δ*cre1*-43 mutants compared to wild type strain to obtain log fold change (logFC), average expression and differential expression status (deg status) for C. cinerea protein IDs.

Table S4: Analysis of Quant-Seq data of Δ*cre1*-91 mutants compared with the wild-type strain to obtain the log fold change (logFC), average expression and differential expression status (deg status) for the *C. cinerea* protein IDs.

Table S5: Normalized count per million reads (CPM) for Δ*cre1*-43, Δ*cre1*-91, and wild-type samples (Sheet 1). Total number of raw reads, trimmed reads, uniquely mapped reads, and assigned reads obtained from Quant-Seq data for each sample. Also shown is the percentage of uniquely mapped reads compared to the raw reads obtained (Sheet 2).

Table S6: The table shows the GO terms along with the corresponding number of genes (annotated, significant) in the up and down-regulated genes of *C. cinerea cre1* mutants. Sheet 1: Shows the GO terms in upregulated genes of *C. cinerea* Δ*cre1*-43 and Δ*cre1*-91 mutants. Sheet 2: Shows the GO terms in down-regulated genes of *C. cinerea* Δ*cre1*-43 and Δ*cre1*-91 mutants. BP - Biological process, MF - Molecular function and CC - Cellular component.

Table S7: Table shows the differentially expressed CAZymes in the *C. cinerea* Δ*cre1*-43 and Δ*cre1*-91 mutants Sheet 1: Shows the PCDWE CAZymes along with their protein IDs, CAZyme classes, plant cell wall components, deg status, and average CPM for the mutants and wild type. Sheet 2: Shows the FCW CAZyme along with their protein IDs, CAZyme classes, fungal cell wall components, deg status and average CPM for the mutants and wild type.

Table S8: The differentially expressed transcription factors (protein IDs) in the Δ*cre1*-43 and Δ*cre1*-91 mutants along with their average CPM values, differential expression status (deg status), InterPro description, and the names of their orthologs in other species.

Table S9: Putative plasma membrane transporters differentially expressed in Δ*cre1*-43 and Δ*cre1*-91 mutants along with their average CPM values, differential expression status (deg status), InterPro ids, InterPro description and probable location in the cell.
